# Network Pharmacology Analysis and *In Vitro* Validation of the Active Ingredients and Potential Mechanisms of Gynostemma Pentaphyllum Against Esophageal Cancer

**DOI:** 10.2174/0113862073280183240108113853

**Published:** 2024-01-12

**Authors:** Jianxin Guo, Zhongbing Wu, Xiaoyue Chang, Ming Huang, Yu Wang, Renping Liu, Jing Li

**Affiliations:** 1 College of Integrated Chinese and Western Medicine, Hebei Medical University, Shijiazhuang 050011, China;; 2 The Fourth Hospital of Hebei Medical University, Shijiazhuang, 05001l, China

**Keywords:** Gynostemma pentaphyllum, esophageal cancer, PI3K/AKT, network pharmacology, molecular docking, KYSE-150

## Abstract

**Background:**

Esophageal cancer (EC) is one of the deadliest malignancies worldwide. Gynostemma pentaphyllum Thunb. Makino (GpM) has been used in traditional Chinese medicine as a treatment for tumors and hyperlipidemia. Nevertheless, the active components and underlying mechanisms of anti-EC effects of GpM remain elusive.

**Objective:**

This study aims to determine the major active ingredients of GpM in the treatment of EC and to explore their molecular mechanisms by using network pharmacology, molecular docking, and *in vitro* experiments.

**Methods:**

Firstly, active ingredients and potential targets of GpM, as well as targets of EC, were screened in relevant databases to construct a compound-target network and a protein-protein interaction (PPI) network that narrowed down the pool of ingredients and targets. This was followed by gene ontology (GO) functional and Kyoto Encyclopedia of Genes and Genomes (KEGG) pathway enrichment analyses. Next, molecular docking, ADME and toxicity risk prediction, cell viability assays, *in vitro* scratch assays, Transwell cell invasion assays, and Western blotting analysis were subsequently applied to validate the results of the network analysis.

**Results:**

The screening produced a total of 21 active ingredients and 167 ingredient-related targets for GpM, along with 2653 targets for EC. The PPI network analysis highlighted three targets of interest, namely AKT1, TP53, and VEGFA, and the compound-target network identified three possible active ingredients: quercetin, rhamnazin, and isofucosterol. GO and EKGG indicated that the mechanism of action might be related to the PI3K/AKT signaling pathway as well as the regulation of cell motility and cell migration. Molecular docking and pharmacokinetic analyses suggest that quercetin and isoprostanoid sterols may have therapeutic value and safety for EC. The *in vitro* experiments confirmed that GpM can inhibit EC cell proliferation, migration, and invasion and suppress PI3K and AKT phosphorylation.

**Conclusion:**

Our findings indicate that GpM exerts its anti-tumor effect on EC by inhibiting EC cell migration and invasion *via* downregulation of the PI3K/AKT signaling pathway. Hence, we have reason to believe that GpM could be a promising candidate for the treatment of EC.

## 1 INTRODUCTION

Esophageal cancer (EC), one of the most fatal malignancies worldwide, is the sixth leading cause of cancer-related deaths [1]. In 2020, it caused 544,076 deaths globally, accounting for 5.5% of all cancer deaths, and 604,100 new cases were diagnosed, representing 3.1% of all cancers [2]. Due to a lack of early symptoms, more than 50% of EC patients have already developed unresectable tumors or metastases visible on imaging by the time of initial diagnosis [3]. These patients are mainly treated with a combination of radiotherapy and/or chemotherapy and preoperative chemoradiation or perioperative chemotherapy [4]. To date, combination chemotherapy with cisplatin and 5-fluorouracil remains the most common treatment for patients with locally advanced unresectable or metastatic EC [5, 6]. However, these radiotherapy drugs often have a variety of adverse effects, resulting in a poor prognosis with an overall five-year survival rate of only 15 – 25% [7, 8]. Since chemically synthesized substances can result in a variety of side effects, there has been growing interest among pharmacologists in phytochemicals, structurally diverse biologically active compounds of plant origin that have important medicinal and nutritional properties [9].

One emerging group of complementary and alternative medicine in cancer treatment belongs to Traditional Chinese Medicine (TCM) [10], whose extracts and derivatives have been shown to be effective against a wide range of cancers [11]. Specifically, Gynostemma pentaphyllum Thunb. Makino (GpM), a Chinese herbal medicine belonging to the Cucurbitaceae family, is used in the treatment of tumors [12]. Recently, there have been reports on the anti-tumor activities of GpM in various types of cancer, including hepatocellular carcinoma, lung carcinoma, colorectal carcinoma [13-15], and renal carcinoma [12]. The herb has been shown to inhibit tumor cell migration and invasion, promote tumor cell apoptosis, and block the tumor cell cycle *via* multiple pathways. In particular, Jingxin Ma *et al.* showed that GpM saponin inhibited the proliferation of EC cells by rendering them more sensitive to chemotherapy [16]. Nevertheless, the active components and underlying mechanisms of anti-EC effects of GpM remain elusive.

A new approach adopted in TCM-based drug discovery is network pharmacology, an emerging discipline based on a multilayer network structure [17]. Network pharmacology identifies targets by constructing graphs linking diseases with drugs [18, 19]. Its capability of identifying multiple possible signaling pathways lends itself as a tool to illuminate the multi-component and multi-target nature of TCM [20]. Capable of analyzing the effects of multi-component drugs on the human body on a systematic level, network pharmacology helps researchers with the identification of therapeutic targets of active drug ingredients, the first step in improving drug efficacy and reducing side effects [21]. Computer simulations to calculate the interactions between drug molecules and target proteins, as well as the absorption, distribution, metabolism, excretion and toxicity (ADME/T) properties of drug molecules, are considered feasible and provide critical information for the development of safe and effective drugs [22, 23].

This study adopted a network pharmacology approach to determine the key targets and molecular mechanisms underlying the effects of GpM against EC. In addition, molecular docking technology was used to assess the interaction of identified active compounds in GpM with potential EC targets, and the results were verified using CCK-8 assays, *in vitro* scratch assays, Transwell assays, and western blot analyses. The design of the current study is illustrated in Fig. (**1**).

## 2 MATERIALS AND METHODS

### 2.1 Ingredient Screening and Drug Target Identification

Potential active compounds were searched in the TCMSP database (https://tcmspw.com/tcmsp.php) [24] and SymMap database (http://www.symmap.org) [25] with the keywords “gynostemma” and “Jiao Gu Lan”. After the removal of duplicates, the search results were screened for results with oral bioavailability (OB) ≥ 30% and drug-likeness (DL) ≥ 0.18 [26]. The list of vetted compounds was uploaded onto the TCMSP website again to obtain a list of potential drug targets, which was then fed into the UniProt database with the constraints “reviewed” and “human” [27] to obtain the standard gene names.

### 2.2 Disease Target Acquisition

The keyword “esophageal cancer” was searched on DisGeNET (https://www.disgenet.org/) [28], DRUGBANK (https://go.drugbank.com/) [29], GeneCards (https://www.genecards.org/) [30], PharmGKB (https://www.pharmgkb.org/about) [31], TTD (https://db.idrblab.net/ttd/) [32], and OMIM (https://omim.org/) [33] to obtain disease target genes. The results were aggregated with duplicated values removed before being loaded into UniProt to obtain their standard gene names.

### 2.3 GpM Compound-target Network Construction

The intersection of the targets found in the previous two steps was obtained using Venny 2.1 (http://jvenn.toulouse.inra.fr/app/example.html) [34]. To visualize the therapeutic characteristics of the identified active ingredients in GpM, a network graph was generated from the common targets and their corresponding ingredients in GpM using Cytoscape 3.9.1. Parameters of the network, such as the degree of each node, were calculated in the software.

### 2.4 Protein-protein Interaction (PPI) Network Construction

A PPI network was constructed from the common targets using STRING database (https://cn.string-db.org/) [35], with species limited to “Homo sapiens” and a minimum interaction score of 0.400. The results, exported as TSV files, were visualized in Cytoscape 3.9.1 with nodes sorted in descending order of degree.

### 2.5 GO and KEGG Enrichment Analyses

The same set of common targets was uploaded as a gene list onto Metascape’s webpage [36] to conduct gene ontology (GO), functional enrichment analyses of biological process (BP), cellular component (CC), and molecular function (MF) and to perform Kyoto Encyclopedia of Genes and Genomes (KEGG) pathway enrichment analysis [37], both for H. sapiens. The ten entries with the highest -LogP in the result list were plotted on ChiPlot (https://www.chiplot.online/).

### 2.6 Molecular Docking

The targets and ingredients of interest identified by the network analyses were assigned as ligands and receptors, respectively, for molecular docking. PDB files of the target proteins were retrieved from the PDB database (https://www.rcsb.org), and SDF files of the ingredient molecules were downloaded from the TCMSP database (https://tcmsp-e.com). In AutoDockTools-1.5.6, receptor proteins were dehydrated and protonated, and ligand molecules were protonated. Active site docking was performed using plug-ins, “autogrid” and “autodock”, with the ligand binding free energy calculated using the Lamarckian genetic algorithm (LGA) algorithm. A binding energy between -5.0 and -7.0 kcal/mol is considered to indicate a fairly good affinity, and one more negative than -7.0 kcal/mol is seen as really good [38]. The results were visualized in PyMOL.

### 2.7 ADME and Toxicity Risk Prediction

The isomeric SMILES of the main active compounds (quercetin, rhamnazin, and isofucosterol) were obtained from Explore Chemistry (https://pubchem.ncbi.nlm.nih.gov/) and imported into SwissADME [39] (http://www.swissadme.ch) and OSIRIS Toxicity software (https://www.organic-chemistry.org/prog/peo/) for prediction of physicochemical properties, pharmacokinetics, and toxicity risk [40, 41]. SwissADME performs a series of tests on the structures of the compounds, including Lipinsky, Ghose, Veber, Egan, PAINS, and Muegge filtration tests. OSIRIS Toxicity software calculates the physicochemical properties (drug similarity and drug scores) and toxicity risks (mutagenicity, tumorigenicity, irritation, and reproductive effects) of the compounds. Essentially, these tests examine the prospects of ligands as future drugs.

### 2.8 *In Vitro* Validation

#### 2.8.1 Materials

Dried GpM herbs were bought from a Tong Ren Tang pharmacy. RPMI 1640 culture medium with L-Alanyl-L-Glutamine (RPMI 1640, C3010-0500) and fetal bovine serum (FBS, C3801-0050) were both purchased from Shanghai XP Biomed Ltd. Human esophageal squamous carcinoma cell lines (KYSE-150) were acquired from The Cell Bank of Type Culture Collection of Chinese Academy of Sciences, Shanghai, China. The rest were all purchased from ABclonal, Wuhan: Trypsin-EDTA, liquid, 25% Trypsin with EDTA (Gibco #25200056), Cell Counting Kit-8 (CCK-8 kit) (Ruipate #RK1028), matrigel basement membrane matrix (Abwbio #0827045), Transwell insert with 8.0 µm pore polyester membrane (BIOFIL #TCS003024), PI3 Kinase p85 antibody(#A22996), Phospho-PI3K p85 Rabbit pAb (#AP0854), AKT Rabbit pAb (#A11016), Phospho-AKT-T308 Rabbit mAb (#AP1332), β-Actin Rabbit pAb (#AC006), Goat Anti-Rabbit IgG /HRP horseradish peroxidase labelled goat anti-rabbit IgG (Ruipate#S1002), and Ultra High Sensitivity ECL Kit (MCE#HY-K1005).

#### 2.8.2 Preparation of the Drug

Weighed GpM herbs were soaked in distilled water for at least 30 min and boiled. The infusion was filtered through gauze, and the filtrate was concentrated down to 100 mL by evaporation in a vacuum. The concentrated filtrate was then freeze-dried to obtain 5.4g frozen powder, which was stored at -80°C in a refrigerator for further use.

#### 2.8.3 Cell Viability Assays

KYSE-150 cell cultures in the log phase were inoculated into individual wells of a 96-well culture plate in GpM solutions of different concentrations (0, 0.4, 0.8, 1.6 and 3.2 mg/mL). Each well initially contained 2000 cells in 100 μL of solution. After either 24h of incubation or another 2h of incubation following the addition of 10 μL of CCK-8 solution per well, the optical density value (OD) of each well was recorded using a microplate reader at 450 nm. Cell viability was calculated using the following equation:


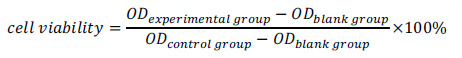


#### 2.8.4 In Vitro Scratch Assays

In each well of a 24-well culture plate, a monolayer of cells was grown from 6×10^4^ EC cells. A scratch line was made in the monolayer with the tip of a 200 μL plastic micropipette tip, and detached cells were washed away with PBS. Immediately afterward, a snapshot of each monolayer was taken under a microscope. Wells in the test group were each replenished with 500 μL media containing GpM solutions at either 0, 0.55 or 1.1 mg/mL, and wells in the blank group were replenished with fresh media of the same volume but without GpM solution. Twenty-four hours later, an image of each monolayer was captured again. Changes in wound area were measured using Image J 1.53K.

#### 2.8.5 Transwell Cell Invasion Assays

The effect of GpM on the invasiveness of EC cells was measured using transwell units with an 8-μm-pore membrane. Transwell inserts were placed into a 24-well culture plate. Then, 200 μL of empty medium cell suspension containing 5×10^5^ cells was added on top of the filter membrane in each transwell insert, and 600 μL of 10% FBS medium (with drug concentrations of either 0, 0.55, or 1.1 mg/mL) was added to each well outside the insert. After 24 hours of incubation, the medium was removed from wells and inserts, and the plate was washed twice with PBS. Non-migrated cells inside the inserts were removed using a cotton swab. The inserts were immersed in 4% paraformaldehyde for 15 min to allow cell fixation and then in 0.1% crystal violet for 20 min for staining. Finally, each transwell insert was imaged under a microscope in different fields of view to get an average number of migrated cells.

#### 2.8.6 Western Blotting Analysis

EC cells were inoculated in three cell culture flasks and incubated for 24 hours with GpM (0, 0.55, or 1.1 mg/mL, respectively) added for stimulation. Cell lysates were prepared using RIPA lysis buffer. The protein concentration of each cell lysate was determined using a BCA protein concentration assay kit. Electrophoresis of lysate samples was run on 10% polypropylene gels, and separated proteins were then transferred onto PVDF membranes, which were blocked with 5% skimmed milk at room temperature. The membranes were then incubated with primary antibodies PI3K (1:5000), P-PI3K (1:1000), AKT (1:800), P-AKT (1:3000), and β-actin (1:800) overnight at 4°C. Following incubation, the membranes were rinsed by TBST before incubation with secondary antibodies (1:5000) at room temperature. Finally, protein bands were detected using an ECL reagent. Images were analyzed using Image J 1.53K to calculate the relative expression of target proteins.

### 2.9 Statistical Analysis

We used SPSS 25.0 and GraphPad Prism 8.0 for statistical analysis. Groups of data that conformed to normal distribution were tested using ANOVA; otherwise, a nonparametric rank sum test was used. *P* < 0.05 was considered statistically significant.

## 3 RESULTS

### 3.1 Ingredient Screening and Drug Target Identification

A total of 21 active ingredients of GpM, along with 167 therapeutic targets, were identified in TCMSP and SymMap databases with OB ≥ 30% and DL ≥ 0.18 (Table **1**).

### 3.2 EC Disease Target Prediction

Our search in DisGeNET, DRUGBANK, GeneCards, PharmGKB, TTD, and OMIM databases with “esophageal cancer” as the keyword resulted in 2653 disease targets in total (after removal of duplicates).

### 3.3 Compound-target network construction

**Fig. (2):**
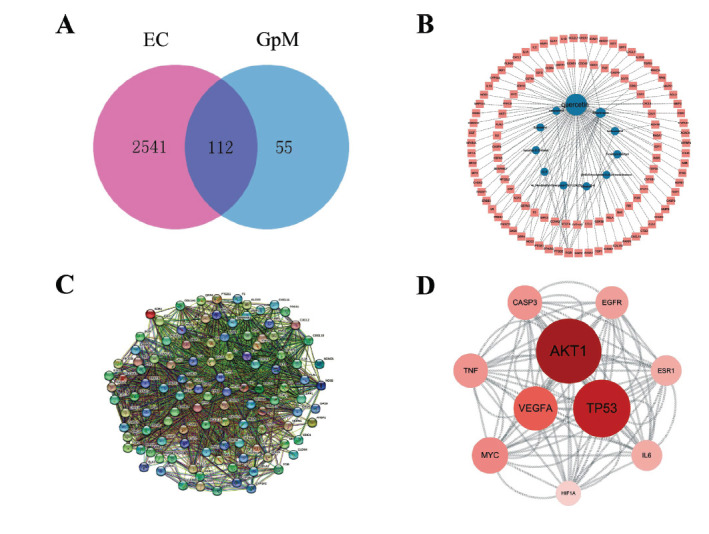
GpM and EC network analyses. (**A**) Venn diagram of GpM and EC targets. (**B**) Compound-target network built from the co-owned targets in (**A**). Targets are represented by the round nodes in the middle, with bigger ones having more connections. (**C**) PPI network generated from the co-owned targets in (A). (**D**) A cluster of key targets retrieved from (**C**), with node size and color being proportional to the degree of interaction.

A Venn diagram constructed from the two sets of targets showed 112 items in the overlapping region (Fig. **2A**), which were the 112 potential targets of GpM in the treatment for EC. A compound-target network generated from these potential targets and their corresponding drug ingredients is shown in Fig. (**2B**). In the network, active ingredients are represented by the blue nodes in the innermost circle, and the potential targets are represented by the pink nodes arranged on the outside. Each edge represents the interaction between an ingredient and a target. The contiguous network consists of 123 nodes and 131 edges. The ingredient nodes with the highest degree were quercetin, rhamnazin, and isofucosterol; in other words, these were likely the top three active ingredients of GpM in EC.

### 3.4 PPI Network of GpM-EC Targets

The 112 common targets of GpM and EC were imported into the STRING database, and the PPI network shown in Fig. (**2C**) was generated, with 112 nodes, 2071 edges, an average of 37 edges per node, and PPI enrichment *p*-value < 1.0×10^-16^. The network, exported as a TSV file, was analyzed with CytoNCA plug-in in Cytoscape to find the 10 nodes (or targets) with the highest degree. These nodes and their connections are illustrated in Fig. (**2D**), in which the size and the color of a node indicate its interconnectivity. Network analysis showed that AKT1, TP53, and VEGFA were the three targets most closely connected to the others.

### 3.5 GO Analysis and KEGG Pathway Enrichment Analysis

The 112 common targets were analyzed with the GO enrichment tool on Metascape. With p < 0.01, the analysis returned 1591 biological processes (BP), 59 cellular components (CC), and 143 molecular functions (MF). The top ten results with the highest -LogP in each category are shown in Figs. (**3A-C**). The list of BPs was topped by “positive regulation of cell migration”, “positive regulation of cell motility”, and “positive regulation of locomotion”; CCs mainly included membrane rafts and components related to transcription, transport, or endocytosis; and the top ten MFs basically narrowed down to DNA-binding transcription factor, RNA polymerase II-specific DNA-binding, kinase binding, cytokine receptor binding, and signal receptor regulator activity.

**Fig. (3):**
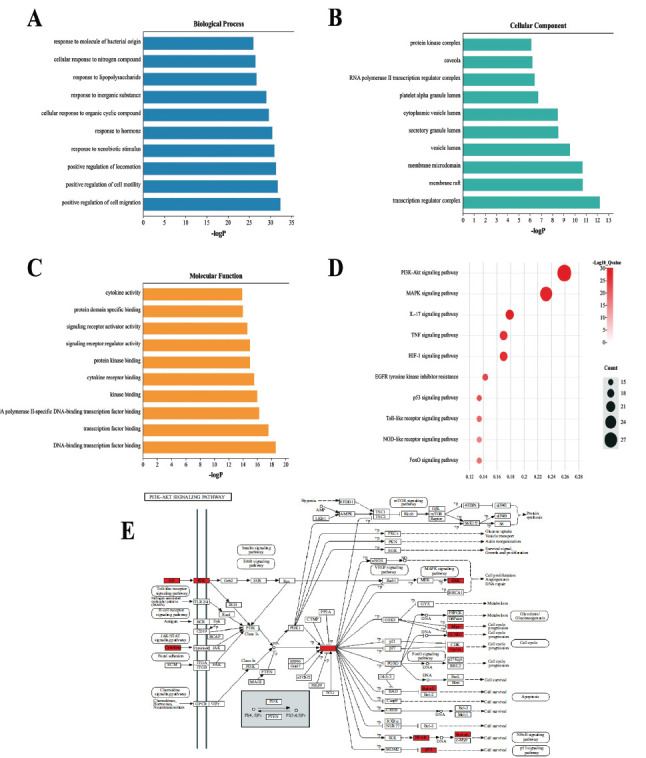
GO and KEGG enrichment analyses of GpM in the treatment of EC. (**A**) GO-BP analysis; (**B**) GO-CC analysis; (**C**) GO-MF analysis; (**D**) KEGG enrichment analysis; (**E**) Distribution of GpM target proteins on the PI3K-AKT pathway. Highlighted nodes are the identified target proteins of GpM for EC treatment, while unhighlighted nodes are irrelevant targets in the pathway.

The KEGG pathway enrichment analysis turned up 188 results at p < 0.01, out of which 10 results associated with tumor and having the highest -LogP were selected. These 10 pathways are shown in Fig. (**3D**) (sorted by target count). Among the ten, the pathways containing the largest number of common targets were the PI3K/AKT signaling pathway, MAPK signaling pathway, and IL-17 signaling pathway, and the one most significantly impacted was the PI3K/AKT signaling pathway (Fig. **3E**).

### 3.6 Molecular Docking

**Fig. (4):**
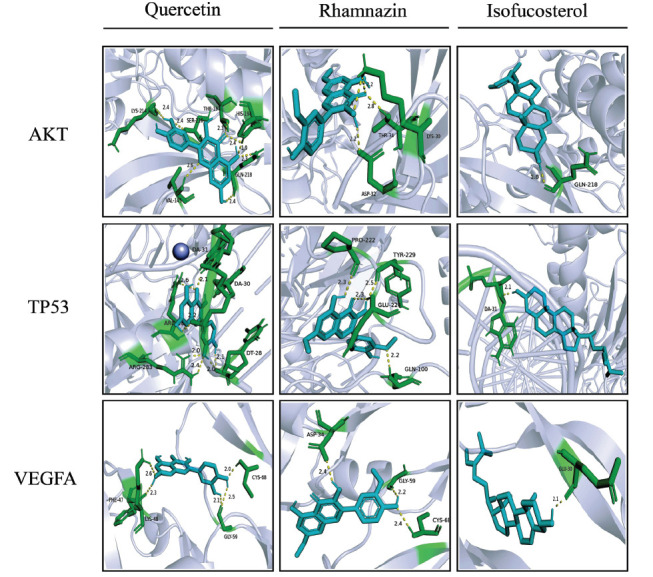
Molecular docking shows the binding of GpM active components to the three targets of interest.

Molecular docking is a method that, among other things, can predict the pose of a TCM compound as it binds to the binding site of a disease-related target. A more negative binding energy between the two indicates a greater binding affinity, suggesting a higher likelihood that the compound can act on the target, altering its structure and function and regulating its associated signaling pathways. In the present study, molecular docking was conducted in AutoDock (Fig. **4**). The molecules chosen were the three active ingredients of interest (quercetin, rhamnazin, and isofucosterol) identified from the compound-target network and the three targets of interest (AKT1, TP53, and VEGFA) identified from the PPI network analysis. In the simulation, all three chosen GpM active ingredients exhibited fairly strong affinity with each of the three targets, as exemplified by negative binding energy (≤-5.0kcal/mol) and by hydrogen bonds that formed between each ligand-receptor pair. In particular, three pairs (isofucosterol with AKT1, isofucosterol with VEGFA, and quercetin with VEGFA) all had binding energy more negative than -8.0kcal/mol (Table **2**). These results suggest that the identified active ingredients and drug targets may be the ones whereby GpM exerts its anti-EC effect.

### 3.7 ADME and Toxicity Risk Prediction

Understanding the physicochemical properties, pharmacokinetics, drug class properties and toxicity risks of compounds is an important step in discovering drugs with better bioavailability and efficiency [42-44]. Pharmacokinetic analysis was conducted in SwissADME, where quercetin, rhamnazin and isofucosterol were evaluated. The results, mentioned in Table **S1**, indicate that all three compounds meet Lipinski's “Rule of Five” [45, 46] and can be passively absorbed from the gastrointestinal tract, though isofucosterol exhibited low absorption efficiency. Results of toxicity risk prediction in OSIRIS (Table **3**) showed that quercetin was highly mutagenic and tumorigenic, rhamnazin was highly mutagenic, and isofucosterol was highly stimulating.

### 3.8 CCK-8 Cell Viability Assays

**Fig. (5):**
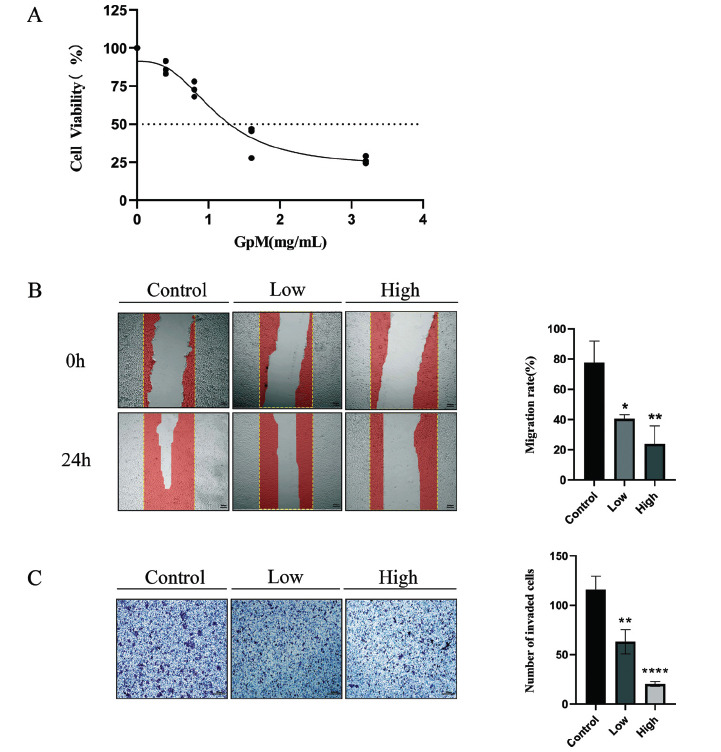
GpM inhibits *in vitro* proliferation, migration, and invasion of KYSE-150 cells in a dose-dependent manner. (**A**) Effects of GpM on cell viability at different concentrations, assessed by CCK-8 assays. (**B**) Effects of GpM on cell migration during wound healing at different concentrations, assessed by scratch assays. (**C**) Effects of GpM on cell invasion at different concentrations, assessed by transwell invasion assays. The data are represented as means ± SD. *P<0.05;**P<0.01;***P<0.001;****P<0.0001.

The responses of KYSE-150 cells to treatment with GpM solution showed that the drug significantly inhibited the proliferation of KYSE-150 cells and that the inhibitive effect was positively correlated with the concentration of the solution used, with an IC_50_ of 1.121mg/mL after 24 hours of treatment (Fig. **5A**).

### 3.9 GpM Inhibited Migration and Invasion of EC Cells

From the scratch assays evaluating the effect of GpM solution on the migration of KYSE-150, we found that GpM reduced cell migration into the wound area in a dose-dependent manner (Fig. **5B**). From the transwell cell invasion assays, we found that the average number of cells that migrated and crossed the membrane was significantly lower in every GpM group than in the control group and that the decrease was more significant in groups with higher GpM dose (Fig. **5C**). Overall, GpM was shown to inhibit KYSE-150 migration and invasion *in vitro* in a concentration-dependent manner.

### 3.10 GpM Downregulated the PI3K/AKT Signaling Pathway

**Fig. (6):**
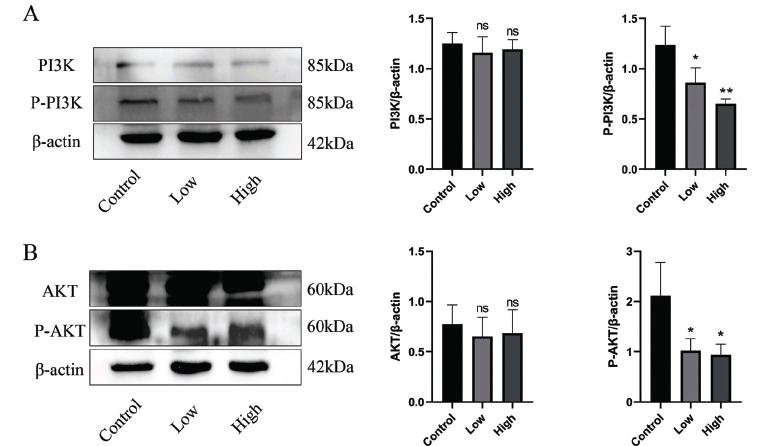
Western blot analysis of the expression of the PI3K/AKT signaling pathway. (**A**) Representative blots showing the effect of low (0.55 mg/mL) or high (1.1 mg/mL) aqueous extract on the expression level of PI3K/AKT signaling pathway in KYSE-150 cells. (**B**) Quantified results of protein levels, which were adjusted to corresponding β-Actin protein level and expressed as fold change relative to control (mean + S.D., n = 3). Differences among all groups were determined by Student's t-test, *p < 0.05, **p < 0.01, as compared with vehicle control.

We measured the expression levels of proteins involved in the PI3K/AKT signaling pathway. The results showed that expression levels of P-PI3K and P-AKT were lower in both GpM-treated groups than in the control group, with the effect being more pronounced in the high group (the group of cell cultures incubated in 1.1mg/mL of GpM solution) (P < 0.01) (Fig. **6**). These results suggest that GpM could inhibit EC cell migration and invasion *via* regulation of PI3K/AKT signaling pathway.

## 4 DISCUSSION

EC is one of the most lethal cancers globally, but so far, drugs for its treatment are still unsatisfactory. Meanwhile, a new approach to drug discovery has emerged in the form of active-ingredient screening of Chinese medicines. Recently, GpM has become the focus of attention for its low pharmacological toxicity and anti-cancer effects [47], for which several mechanisms have been suggested. A study by Xing S-F found that in human lung cancer cell lines, GpM induces apoptosis, blocks the cell cycle at the early G0/G1 phase, and inhibits migration [15]. A more recent study concluded that GpM triggers apoptosis in renal cancer cells by regulating the PI3K/AKT/mTOR signaling pathway [12]. Despite the attention, what no individual study has achieved is to paint a full picture of the myriad mechanisms of GpM. Nevertheless, this gap can be filled by leveraging the power of network pharmacology, which excels at uncovering intricate drug-disease interactions and their underlying mechanisms at a systematic level [48]. The present study adopted this systematic approach to elucidate the therapeutic effects of GpM on EC and validated the results using molecular docking and *in vitro* experiments.

First, network pharmacology screening uncovered 21 potential active ingredients of GpM, including quercetin, rhamnazin, and isofucosterol. Quercetin belongs to the flavonol group of compounds, and it has been shown to have antitumor effects on many human cancer cell lines, such as prostate, lung, colon, cervical and breast cancers [49, 50]. For example, quercetin has been reported to inhibit the migratory and invasive abilities of colon 26 cells, possibly through regulation of the expression of matrix metalloproteinases (MMPs) and tissue inhibitors of metalloproteinases (TIMPs) [51]. In addition, flavonols were previously reported to inhibit NF-κB pathway activation and upregulate the cytoprotective nuclear factor erythroid 2-related factor 2 (NRF2) cascade response [52].

Another study proposed that mechanisms of quercetin in treating colon cancer may involve inhibition of the TLR4/NF-κB signaling pathway as well as the reduction in the production of inflammation factors, including TNF-α, Cox-2, and IL-6 [53]. Interestingly, in patients with gastrointestinal tumors, it has been found that the expression of genes, such as TLRs [54, 55], WFDC2, TTLL12, THRA, and EPHB3 [56], which can trigger an immune response and release of pro-inflammatory factors [57] and thus accelerate tumor cell invasion, is disturbed. Rhamnazin, one of the other ingredients identified, has also been shown to have or enhance anti-tumor effects in lung cancer and hepatocellular carcinoma [58, 59].

Then, of the 112 common targets of GpM and EC, three were identified in PPI enrichment as the most important: AKT1, TP53, and VEGFA. It has been shown that in colorectal cancer, collagen type XI alpha 1 (COL11A1) [60] with histone deacetylase 9 (HDAC9) [61] affects the PI3K/AKT and TP53 signaling pathways, leading to uncontrolled tumor invasion.

AKT1, a serine/threonine kinase belonging to the AKT subfamily, is a well-characterized effector of PI3K in the PI3K/AKT/mTOR signaling pathway, and its dysregulation is implicated in many types of human cancers [62]. AKT1 is involved in several biological processes, such as tumor cell migration, cell proliferation, and apoptosis [63]. P53 is involved in tumor suppression [64], a process believed to be mediated by inducing apoptosis, cell cycle arrest, and senescence [65]. Vascular endothelial growth factor (VEGF) is a family of proteins important in angiogenesis; VEGFA, a member of the VEGF family, plays a key role in regulating angiogenesis. VEGFA is often dramatically upregulated in most solid tumors, an effect that results in the proliferation of endothelial cells and the formation of new blood vessels [66, 67].

KEGG pathway enrichment analysis showed that GpM treatment of EC mainly involved the PI3K-AKT signaling pathway, MAPK signaling pathway, and IL-17 signaling pathway, all of which are associated with tumor diseases. Based on the correlation of each pathway in the result of the enrichment analysis, we chose the PI3K-AKT signaling pathway as the potential candidate for further analysis. In fact, mounting evidence suggests that the PI3K-AKT pathway may play an important role in tumor cell proliferation, autophagy, apoptosis, and angiogenesis [68]. Also, the PI3K-AKT signaling pathway has been linked to distant metastasis and chemotherapy resistance and has been found to promote epithelial-mesenchymal transition (EMT) in human cancer cells [69, 70]. Results of GO functional enrichment analysis pointed to cell motility and migration as the main biological processes and lipid rafts as a likely cellular location involved in GpM treatment of EC. Lipid rafts have been shown to be connected with the PI3K-AKT signaling pathway and play an important role in cancer metastasis [71].

Subsequently, we performed molecular docking simulations to study the interaction between the three ingredients of interest and the three target proteins of interest. Of the nine docking combinations, three had binding energy more negative than -8.0 kcal/mol, indicating strong affinity. The active ingredients that showed up in these three combinations were isofucosterol and quercetin. Isofucosterol has been known for its lipase inhibitory activity and as a potential anti-obesity agent [72], but we have found no report of its possible anti-cancer actions.

In addition, ADME and toxicity risk prediction results showed that quercetin, rhamnazin and isofucosterol are well absorbed by the gastrointestinal tract but do not pass through the BBB and, therefore, have no central nervous system (CNS) side effects [73]. None of the three compounds had reproductive effects, and quercetin and rhamnazin were mutagenic. The mutagenicity of quercetin was suggested in the 1970s [74], but more recent studies [75] have shown that of the four common flavonoids (luteolin, apigenin, quercetin, and genistein), quercetin is the least mutagenic. The International Agency for Research on Cancer (IARC) concluded that quercetin is not a human carcinogen [76] and that quercetin has significant cancer inhibitory effects [77-79]. In summary, quercetin has the most potential to be a drug among the three main active ingredients of GpM for EC inhibition.

Results of the *in vitro* experiments showed that GpM inhibited the proliferation, migration, and invasion of KYSE-150 cancer cells. Levels of P-AKT and P-PI3K expression were significantly lower in GpM-treated cells than in untreated cells, while total PI3K and AKT levels were not affected. Taken together, these results suggest that the PI3K/AKT signaling pathway was inhibited by GpM compounds (quercetin, rhamnazin and isofucosterol) in a dose-dependent manner.

Given the unsatisfactory efficacy of current treatments of EC, it is imperative for new therapeutic strategies to be developed. We propose isofucosterol and quercetin as drug leads for EC treatment and AKT1, P53, and VEGFA as potential drug targets.

The limitation of this study is a lack of *in vivo* experiments to further add credibility to the conclusions. Nevertheless, other mechanisms of action of GpM on esophageal cancer, such as induction of autophagy, apoptosis, and blockage of the cell cycle, should probably be explored in silico first before investing in *in vivo* tests.

## CONCLUSION

Our findings indicate that GpM exerts its anti-tumor effect on EC by inhibiting EC cell migration and invasion *via* downregulation of the PI3K/AKT signaling pathway. Hence, we have reason to believe that GpM could be a promising candidate for the treatment of EC.

## Figures and Tables

**Fig. (1):**
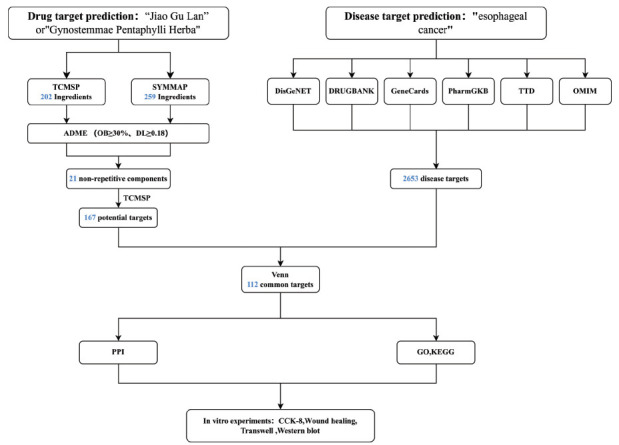
Flow chart of the research conducted in the present study.

**Table 1:** Potential active ingredients of GpM.

**CAS**	**Molecule Name**	**MW**	**OB (%)**	**DL**
73123-10-1	Quercetin	302.25	46.43	0.28
552-54-5	Rhamnazin	330.31	47.14	0.34
18472-36-1	Isofucosterol	412.77	43.78	0.76
83-46-5	Sitosterol	414.79	36.91	0.75
81474-82-0	Gypenoside XXVII_qt	418.73	30.21	0.74
80356-14-5	CLR	386.73	37.87	0.68
80321-64-8	Gypenoside XII	785.14	36.43	0.25
90058-53-0	Gypenoside XXXV_qt	444.77	37.73	0.78
6859-20-7	Ruvoside_qt	390.57	36.12	0.76
62025-49-4	Ginsenoside f2	785.14	36.43	0.25
55866-28-9	4α,14α-dimethyl-5α-ergosta-7,9(11),24(28)-trien-3β-ol	424.78	46.29	0.76
90058-52-9	Gypenoside XXXII	787.11	34.24	0.25
481-18-5	Spinasterol	412.77	42.98	0.76
474-62-4	Campesterol	400.76	37.58	0.71
446-71-9	3'-methyleriodictyol	302.3	51.61	0.27
33644-13-2	(24S)-Ethylcholesta-5,22,25-trans-3beta-ol	410.75	46.91	0.76
2241-90-9	Cyclobuxine	386.69	84.48	0.7
187277-03-8	Gypentonoside A_qt	472.78	36.13	0.8
121108-99-4	Cucurbita-5,24-dienol	426.8	44.02	0.74
110282-46-7	Gypenoside LXXIX	785.14	37.75	0.25
110261-97-7	Gypenoside LXXIV	801.14	34.21	0.24

**Table 2:** GpM active ingredients and key targets binding energy.

	**Binding Energy/(kcal/mol)**		
**Active Ingredient of Interest**	**Quercetin**	**Rhamnazin**	**Isofucosterol**
AKT	-6.13	-5.69	-8.08
TP53	-5.54	-5.13	-7.34
VEGFA	-8.57	-6.33	-8.57

**Table 3:** OSIRIS toxicity software detects potential toxicity risks of the main active ingredients of GpM against EC.

**Drugs**	**DL**	**DS**	**MUT**	**TUMO**	**IRRI**	**REP**
Quercetin	1.6	0.3	Highly	Highly	None	None
Rhamnazin	1.17	0.45	Highly	None	None	None
Isofucosterol	-6.28	0.08	None	None	Highly	None

## Data Availability

The data and supportive information are available within the article.

## LIST OF ABBREVIATIONS

GP: Gene Ontology
PPI: Protein-Protein Interaction
EC: Esophageal Cancer
ADME/T: Absorption, Distribution, Metabolism, Excretion and Toxicity
BP: Biological Process
CC: Cellular Component
KEGG: Kyoto Encyclopedia of Genes and Genomes
OD: Optical Density
MMPs: Matrix Metalloproteinases
TIMPs: Tissue Inhibitors of Metalloproteinases
VEGF: Vascular Endothelial Growth Factor
NRF2: Nuclear Factor Erythroid 2-Related Factor 2
COL11A1: Collagen Type XI Alpha 1
HDAC9: Histone Deacetylase 9
EMT: Epithelial-Mesenchymal Transition
