# Supplementary material for: Network Pharmacology Analysis and In Vitro Validation of the Active Ingredients and Potential Mechanisms of Gynostemma Pentaphyllum Against Esophageal Cancer
Source: Comb Chem High Throughput Screen. 2024 Jan 12;28(3):500–13. doi: 10.2174/0113862073280183240108113853 (PMC12174898; doi:10.2174/0113862073280183240108113853)
Supplement: Supplementary file 1 [file CCHTS-28-3-500_SD1.pdf]

Supplementary Material

Network Pharmacology Analysis and In Vitro Validation of the Active Ingredients and Potential Mechanisms of Gynostemma Pentaphyllum Against Esophageal Cancer

Jianxin Guo[1], Zhongbing Wu[1], Xiaoyue Chang[1], Ming Huang[1], Yu Wang[1], Renping Liu[1] and Jing Li[1,2,*]

[1]*College of Integrated Chinese and Western Medicine, Hebei Medical University, Shijiazhuang 050011, China;* [2]*The Fourth Hospital of Hebei Medical University, Shijiazhuang 05001l, China*

Table 1. Pharmacokinetic characterization of the main active ingredient of GpM against EC as detected by SWISS ADME.

SWISS ADME profile parameters:	Quercetin	Rhamnazin	Isofucosterol
Canonical SMILES	Oc1cc(O)c2c(c1)oc(c(c2=O)O)c1ccc(c(c1)O)O	COc1cc(O)c2c(c1)oc(c(c2=O)O)c1ccc(c(c1)OC)O	CC=C(C(C)C)CCC(C1CCC2C1(C)CCC1C2CC=C2C1(C)CCC(C2)O)C
Molecular Formula	C15H10O7	C17H14O7	C29H48O
Molecular weight	302.24	330.29	412.69
#Heavy atoms	22	24	30
#Aromatic heavy atoms	16	16	0
Fraction Csp3	0	0.12	0.86
#Rotatable bonds	1	3	5
#H-bond acceptors	7	7	1
#H-bond donors	5	3	1
MR	78.03	86.97	132.75
TPSA	131.36	109.36	20.23
iLOGP	1.63	2.81	5.06
XLOGP3	1.54	2.19	8.85
WLOGP	1.99	2.59	7.94
MLOGP	-0.56	-0.07	6.62
Silicos-IT Log P	1.54	2.59	6.88
Consensus Log P	1.23	2.02	7.07
ESOL Log S	-3.16	-3.56	-7.64
ESOL Solubility (mg/ml)	2.11E-01	9.04E-02	9.36E-06
ESOL Solubility (mol/l)	6.98E-04	2.74E-04	2.27E-08
ESOL Class	Soluble	Soluble	Poorly soluble
Ali Log S	-3.91	-4.12	-9.16
Ali Solubility (mg/ml)	3.74E-02	2.50E-02	2.86E-07
Ali Solubility (mol/l)	1.24E-04	7.58E-05	6.92E-10

Ali Class	Soluble	Moderately soluble	Poorly soluble
Silicos-IT LogSw	-3.24	-4.63	-5.83
Silicos-IT Solubility (mg/ml)	1.73E-01	7.71E-03	6.16E-04
Silicos-IT Solubility (mol/l)	5.73E-04	2.33E-05	1.49E-06
Silicos-IT class	Soluble	Moderately soluble	Moderately soluble
GI absorption	High	High	Low
BBB permeant	No	No	No
Pgp substrate	No	No	No
CYP1A2 inhibitor	Yes	Yes	No
CYP2C19 inhibitor	No	No	No
CYP2C9 inhibitor	No	Yes	No
CYP2D6 inhibitor	Yes	Yes	No
CYP3A4 inhibitor	Yes	Yes	No
log Kp (cm/s)	-7.05	-6.76	-2.53
Lipinski #violations	0	0	1
Ghose #violations	0	0	3
Veber #violations	0	0	0
Egan #violations	0	0	1
Muegge #violations	0	0	2
Bioavailability Score	0.55	0.55	0.55
PAINS #alerts	1	0	0
Brenk #alerts	1	0	1
Leadlikeness #violations	0	0	2
Synthetic Accessibility	3.23	3.41	6.15
